# Post translational modification of Parkin

**DOI:** 10.1186/s13062-017-0176-3

**Published:** 2017-02-21

**Authors:** Joy Chakraborty, Valentina Basso, Elena Ziviani

**Affiliations:** 1 Department of Biology, University of Padova, Via Ugo Bassi 58b, 35131 Padova, Italy; 2 Istituto IRCCS San Camillo, Lido di Venezia, Venezia,, Italy

**Keywords:** Parkinson’s disease, Parkin, Post translational modifications, Ubiquitination, Phosphorylation

## Abstract

**Abstract:**

Mutations in the gene encoding for the E3 ubiquitin ligase Parkin are associated to a rare form of familiar autosomal recessive Parkinsonism. Despite decades of research on the Parkin protein, whose structure has been recently solved, little is known about the specific signalling pathways that lead to Parkin activation. Parkin activity spans from mitochondria quality control to tumor suppression and stress protection; it is thus tempting to hypothesize that the broad impact of Parkin on cellular physiology might be the result of different post translational modifications that can be controlled by balanced opposing events. Sequence alignment of Parkin from different species indicates high homology between domains across Parkin orthologs and identifies highly conserved amino acid residues that, if modified, impinge on Parkin functions. In this review, we summarize findings on post translational modifications that have been shown to affect Parkin activity and stability.

**Reviewers:**

This article was reviewed by Prof. Dr. Konstanze F. Winklhofer and by Prof. Thomas Simmen. Both reviewers have been nominated by Professor Luca Pellegrini.

## Background

Parkinson’s Disease (PD) is the second most common neurodegenerative disorder affecting primarily the survival of a specific subset of dopaminergic neurons residing in the *Substantia Nigra Pars Compacta* of the midbrain [1]. Most PD cases are sporadic in origin. However, a small proportion of PD cases derive from mutations in PD associated genes, which have been mainly identified by characterizing familiar Mendelian inherited PD forms [2, 3]. The discovery of these genes (S, PINK1, Parkin, DJ1, LRRK2, VPS35, FBXO7, PLA2G6 and ATP13A2) (see [4] for a review), has greatly enhanced our understanding of the neurodegenerative pathways leading to dopaminergic neurons loss. Although there seem to be various causes of PD, genetic and sporadic forms are almost undistinguishable in terms of specific hallmarks, which at the cellular level includes formation of intracellular inclusions named Lewy Bodies, mitochondria abnormalities and selective loss of DA neurons, leading to the well characterized locomotor impairments at the systemic level [1]. The reason for studying genetic mutations of PD is the belief that the similarities between the sporadic and the inherited forms share a common mechanism of neurodegeneration, which can be more easily dissected at the molecular level in the genetic forms.

## The pleiotropic protein Parkin

Although there are no unequivocally accepted scientific data that explain the selective neurodegeneration of dopaminergic neurons, the pathogenesis of PD appear to converge on three common features: mitochondria dysfunction, oxidative stress and proteins misfolding and aggregation [5]. Indeed studies on Parkin (PARK2), an E3 ubiquitin ligase, which mutations have been associated to the early onset of autosomal recessive Parkinsonism [6], have provided evidences for a direct role of mitochondrial dysfunction in the onset of the disease by regulating the mitochondria quality control via mitophagy. In the mitophagy process, Parkin is selectively recruited to depolarised mitochondria by PINK1 [7], a mitochondrial serine/threonine kinase, also a PD related protein [8]. In healthy mitochondria PINK1 is imported into mitochondria by the TOM/TIM translocase complex, cleaved and rapidly degraded by the proteasome [9–11]. However, on depolarised mitochondria PINK1 remains stable on the surface of mitochondria where it mediates the phosphorylation of Parkin, Parkin substrates and ubiquitin [7, 12–15]. Primed phospho-ubiquitin is specifically used by Parkin to ubiquitinate its targets on the outer mitochondrial membrane (OMM) [16], leading to the recruitment of downstream cytosolic receptors that are required for the activation of autophagy, including p62/SQSTM1, HDAC6, NDP52 and Optineurin [17–20].

Parkin leads to the ubiquitination of a broad number of targets that are expressed on the OMM, among others TOM20, Mitofusins, VDAC and Fis1 [16, 20–22]. Moreover, by targeting proteins with ubiquitin molecules, Parkin plays a crucial role in the degradative pathways mediated by the ubiquitin–proteasome system (UPS), which is required for both clearance of misfolded proteins and stress-induced mitophagy [19]. The available literature suggests that Parkin mediated mitochondrial outer membrane protein ubiquitination recruits proteasome complex to mitochondria, in turn causing rupture of the outer membrane, thus exposing inner membrane proteins which can interact with LC3 and guides mitochondria to mitophagy [23, 24]. It is possible that widespread ubiquitination of mitochondrial surface proteins by Parkin, acts as a general signal for mitochondrial quality control. In that respect, Parkin substrate specificity is debatable because no single Parkin substrate is essential for mitophagy and mitochondrial localization of a number of deubiquitinating enzymes (DUBs), including USP30 and USP2 [25], is sufficient to inhibit mitophagy, where DUBs like USP30 or USP15 knock down [26, 27] rescues defective mitophagy even in the absence of Parkin.

In addition to its established role in mitophagy and UPS, Parkin impacts other neuroprotective cellular pathways [28], including TNFα signaling [29, 30], and Wnt/β catenin signaling [31]. Parkin is also a putative tumor suppressor [32–34]. Interestingly, many of these pleiotropic functions of Parkin, which are dependent on its E3 ubiquitin ligase activity, do not result in ubiquitin dependent degradation of Parkin targets [35–38].

## The power of ubiquitination

How does Parkin fulfill its many biological functions? Recent studies proposed ubiquitination as a modulator of protein activity, via regulating its subcellular localization and its ability to interact with other proteins [39, 40]. In the ubiquitination process, multiple lysine residues on the target protein can be ubiquitinated to produce multi-monoubiquitination [41]. Alternatively, an ubiquitin chain can form upon linear ubiquitination, in which the carboxy-terminal glycine of one ubiquitin molecule form a peptide bond with the amino-terminal methionine of another (linear or M1-linked ubiquitination) [42, 43]. Also, following addition of a single ubiquitin molecule to the target protein, further ubiquitin molecules can be added to the first ubiquitin molecule via linkage on lysine residue, producing an ubiquitinated chain (polyubiquitination). Ubiquitin itself contains seven lysine residues (Lys 6, Lys 11, Lys 27, Lys 29, Lys 33, Lys 48 and Lys 63), which allows the generation of ubiquitin chains with different orientations. Although some chain-types (specifically, K48 and K63-chains [44]) are more common than others, all possible linkages have been detected in cells [45]. Interestingly, the type of ubiquitin chain attached impinges on chain conformation and impacts the physiological outcome. For example, Lys 48 and Lys 11-linked chains adopt compact conformation and target proteins to the 26S proteasome [46–50]. Lys 63-linked chains or monoubiquitination, on the other hand, are the post-translational modifications that regulate lysosome dependent degradation [51, 52]. Monoubiquitination, Lys 63-linked chains and linear ubiquitination have also been described as non-degradative ubiquitination, which can control protein-protein interaction, protein subcellular localization and protein activity [53–59]. Very little is known regarding the physiological relevance of Lys 6, Lys 27, Lys 29 and Lys 33-linked chains and which biological outcomes these modifications would lead to. In that respect, ubiquitin ligases are extremely versatile enzymes that can potentially control almost every cellular process.

It is known that Parkin can promote both degradative Lys 48-mediated ubiquitination and non classical, proteosome-independent ubiquitination, including Lys 6, Lys 11, Lys 63, mono ubiquitination and linear ubiquitination [30, 60, 61]. Although Parkin dependent “regulative” ubiquitination has only recently started to be specifically addressed, emerging evidences suggest that Parkin activity necessarily includes “functional” ubiquitination and has the potential of controlling a broad subset of cellular processes depending on the activating stimuli. Not surprisingly, Parkin exists in an inactive state and it is normally repressed under basal conditions by several mechanisms of autoinhibition.

## Mechanisms of Parkin autoinhibition

**Fig. 1:**
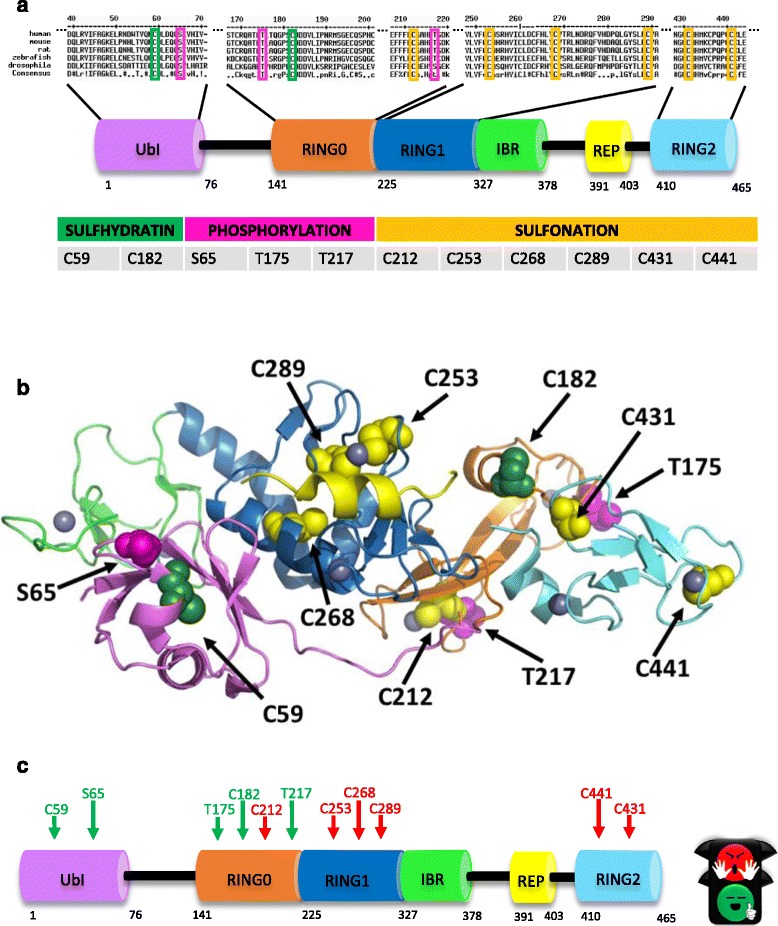
Mapping of Parkin Post translational modifications. **a** Domain architecture of Parkin protein and sequence alignment from different species. We differently *highlighted* the amino acids that are post-translationally modified (*green*: sulfhydration; *pink*: phosphorylation; *yellow*: sulfonation). Parkin consists of *five domains*: *UBL*, ubiquitin-like domain; *RING*, really interesting new gene; *IBR*, in between *RING*; *REP*, repressor element of Parkin. **b** Schematic representation of the full-length structure mapping the post-translational modifications onto the structure. **c** *Primary* structure and domains of Parkin mapping the activating (“*green*”) and inactivating (“*red*”) post-translational modifications

High resolution Parkin crystal structure gave insight into the mechanisms of Parkin autoinhibition. Parkin belongs to the RBR (RING-between-RING) type of E3 ubiquitin ligases, also known as RING/HECT hybrids, consisting of an ubiquitin like domain (Ubl), followed by two RING fingers domains (RING0 and RING1), an in between RING finger domain (IBR), a linker domain called Repressor Element of Parkin (REP) and a third RING finger domain called RING2 [62–65] (Fig. 1a). In the ubiquitin process, ubiquitin-activating enzymes (E1s) activate the C-terminus of the ubiquitin molecule and pass it to E2 conjugating enzymes that accept the activated ubiquitin and coordinate with E3 ubiquitin ligases to finally transfer ubiquitin to the amino group of a substrate protein [66–68]. Ubiquitin coordination and transfer is allowed by forming a thioester bond between catalytic cysteine residues on the E ubiquitin enzymes and the C-terminal carboxyl group of ubiquitin. Parkin can selectively interact and coordinate with different E2 ubiquitin ligase to ubiquitinate its substrates. Interestingly, these E2s are selectively expressed in specific subcellular compartments. For example, Ubc6 and Ubc7 are endoplasmic reticulum-associated E2s that specifically interact with Parkin. Parkin can also interact with E2 enzymes UbcH7, UbcH8 and UbcH13/Uev1. Depending on which E2 enzyme Parkin couples with, different types of ubiquitin modification can arise, resulting in different biological outcomes. For example, Parkin can employ UbcH7 E2 ligase for K48-linked proteasome dependent polyubiquitination, and UbcH13/Uev1a for K63-linked autophagy dependent polyubiquitination [69–73]. How Parkin can choose to couple with a specific E2 ubiquitin ligase is largely unknown.

Two mechanisms of auto inhibition control Parkin activity. Fist, the RING1 domain that contains the binding site for the E2 ubiquitin-conjugating enzyme is occluded by the Ubl and the REP domains. Second, the catalytic site in the RING2 domain is blocked by the RING0 domain. Notably, Parkin catalytic Cys 431 in the RING2 domain is physically distant from the E2 conjugating site, which further suggest that Parkin needs to undergo a conformational change in order to function [62–65]. Therefore, under basal conditions, Parkin maintains a close structure that resembles that of a coiled snake [63] and its ubiquitin ligase activity is repressed (Fig. 1b). Not surprisingly, disease-associated mutations disrupt these interactions.

## Post translational modifications of Parkin

Post translational modifications have emerged as a powerful tool to modulate proteins activity, via regulation of their subcellular localization and ability to interact with other proteins to form signaling complexes. Most Post translational modifications, such as phosphorylation, acetylation, ubiquitination, are reversible modifications, mediated by large families of opposing enzymes. Balanced opposing events mediated by antagonistic enzymes might provide a potential molecular switch to modulate Parkin activity upon specific stimulation. Accordingly, Post translational modifications are required to trigger Parkin activity or to keep it repressed.

In the following sections, we summarize the so far reported Post translational modifications affecting Parkin activity and stability. Sequence alignment of Parkin indicates high homology between domains across Parkin orthologs and identifies highly conserved amino acid residues, most of which are post translationally modified from mammals to insects to impinge on Parkin functions. This analysis highlights evolutionarily conserved posttranslational processes, in relation to Parkin activation, which is not characterized yet.

## Phosphorylation

There are multiple proteins, which are involved in Parkin phosphorylation, PINK1 being the most studied [74–79]. It was first reported by Kim et al. [78] that Parkin activity and mitochondrial localization is PINK1 kinase activity dependant. Authors further described that RING1 and REP domains are indispensable for PINK1 mediated mitochondrial translocation of Parkin, as well as PINK1 dependent phosphorylation of Thr 175/Thr 217 is crucial for its translocation. In a separate study [79] the same was confirmed, and it was further shown that phosphorylation of Parkin is required for Parkin to interact with E2 ubiquitin ligase UbcH13/Uev1a to mediate K63-linked polyubiquitination of IKKγ in NFκB stress response pathway.

Later, it was reported by two simultaneous studies [80, 81] that the PINK1-dependent phosphorylation of Ser 65 of the Ubl domain is required for Parkin translocation as well as stress-induced mitophagy. These findings were confirmed in a subsequent study that used as read out the degradation of Miro1, a *bona fide* Parkin substrate, upon expression of full-length wild type or PD disease-associated Parkin mutants [82]. In vivo data from *Drosophila* also confirmed these findings and additionally showed that phosphorylation of Parkin regulates spontaneous dopamine release from the neuron terminals, flight activity as well as survival of the flies [83]. 

One recent study deciphered the sequence of Parkin activation, and demonstrates that upon mitochondrial depolarization PINK1 phosphorylates ubiquitin at Ser 65, which goes to bind Parkin and Parkin substrates. Primed phospho-ubiquitin makes Parkin more accessible for PINK1 mediated Ser 65 phosphorylation [84]. So, in a nutshell, it has become evident that phosphorylation by PINK1 is the central point of Parkin activation and target recognition [81, 84, 85].

Phosphorylation is not only required for Parkin activation. Quite intriguingly, phosphorylation of Parkin by cyclin dependent kinase 5 (Cdk-5), casein kinase 1 (CK-1) and c-Abl modulates Parkin folding and/or activity [74–76, 86]. Phosphorylation by both Cdk-5 on Ser 131 and CK-1 on Ser 101, Ser 127 and Ser 378 influence the solubility of the protein, leading to increased Parkin aggregation [75]. Quite surprisingly, in both cases Parkin phosphorylation does not seem to particularly affect Parkin E3 ubiquitin activity. The role of c-Abl in regulating Parkin activity was reported by two different studies where the authors found that phosphorylation of Parkin by c-Abl at Tyr 143 can inactivate its E3 ligaes activity [74, 86] in human cell lines and MPTP treated mice. Consistent with this, increased protein levels of c-Abl and tyrosine phosphorylation of Parkin was reported in human post mortem brains from PD patients.

## Ubiquitination

As previously mentioned, poly ubiquitination of proteins in general is the signal for proteasomal degradation. Parkin, though an E3 ubiquitin ligase itself, faces the same fate when multiple ubiquitin chains are attached to it. It has been reported that Parkin mediates its own ubiquitination via K48 proteosome dependent ubiquitin chain formation [87], thus impinging on its own protein turnover. Co-localization of ubiquitinated Parkin and Lewy body in PD patients brain might ignite the idea that ubiquitinated Parkin is a inactive form of the enzyme. However, in reality Parkin mono-ubiquitination at different sites can activate the enzyme. Mutations causing ubiquitination of the Ubl domain of Parkin makes the enzyme constitutively active and thus supports the idea of ‘regulative” Parkin autoubiquitination [88]. One intriguing possibility is that Parkin dependent non-degradative self-ubiquitination might be required to regulate Parkin subcellular localization and its interaction with specific E2 ubiquitin ligases and/or Parkin substrates. In that context, further studies are required to identify the precise site of Parkin ubiquitination and dissect the functional role of specific site ubiquitination.

Consistent with the hypothesis that Parkin self-ubiquitination is a functional ubiquitination, Parkin autoubiquitination is subjected to tight regulation by other factors. Deubiquitinating enzymes (DUBs), for example, antagonize Parkin autoubiquitination [89]. Durcan and colleagues identified DUB Ataxin-3 as a binding partner of Parkin, which interacts with both the Ubl and IBR-RING2 domain of Parkin and promotes Parkin de-biquitination. Mutant Ataxin-3, which polyglutamine expansion is associated with the onset of Machado-Joseph neurodegenerative disease, promotes the degradation of Parkin via autophagy and leads to decreased Parkin levels in vivo [90, 91]. In a subsequent study, the same group showed that Ataxin-3 in fact binds to and coordinate with E2 ubiquitin ligase Ubc7 rather than Parkin, and promotes Parkin de-ubiquitination only upon Parkin autoubiquitination [92].

Recently, the same group has reported that DUB USP8 preferentially remove K6 linked Ub conjugates from Parkin and USP8 silencing hindered Parkin recruitment to depolarised mitochondria, suggesting that USP8 is required for active mitophagy [93].

Overall, these works highlighted an intricate regulation of Parkin ubiquitination that involves the coordinated activities of Parkin, DUBs and E2 ubiquitin ligases. It is tempting to hypothesis that such complex interplay is required to prime Parkin for further Post translational modifications that affect Parkin activity via regulation of its subcellular localization and/or interaction with specific E2 and/or substrates.

## Sumoylation and Neddylation

Post-translational modification of proteins by small ubiquitin like modifiers (SUMO) or in general SUMOylation is still not fully unravelled, though holds the indications that like ubiquitination or phosphorylation, it might have far reaching implications as well. In a very simplistic way, SUMO gets matured, activated and attached to target proteins by a series of specific enzyme complexes, much like the ubiquitination process [94]. Interestingly, from the point of view of PD, three of the prominent proteins that are mutated in familiar Parkinsonism, SNCA, DJ-1 and Parkin, fall under the targets of SUMOylation [95–98]. At first sight, it appears that reports connecting SUMOylation of SNCA and aggregate formation followed by cell death are contradictory [97, 99], but the precise site of SUMOylation might be the deciding factor for aggregate formation and cell death by α-synucleinopathy [100]. As far as Parkin is concerned, reports are significantly less which properly deciphers the physiological role of SUMO attachment to Parkin. One solitary report by Um and Chung [98] demonstrates that covalent attachment of SUMO-1 (but not SUMO-2, which possible due to differential preference of subtrates by SUMO-1, 2 and 3) to Parkin, both in vitro and in vivo, increases its nuclear localization and auto-ubiquitination. In the nucleus, Parkin transcriptionally represses p53 by interacting with p53 promoter. Interestingly, this activity of Parkin is independent of its ligase function.

NEDD8 is another protein that shows similarity with ubiquitin, in terms of structural homology and the way of getting attached to other proteins as post-translational modification [101]. Like ubiquitin, NEDD8 is also expressed in most tissue types and strikingly concentrated in different types of protein aggregates, which includes Mallory bodies in liver, Rosenthal fibres in astrocytoma, neurofibrillary plaques of Alzheimer’s disease and Lewy bodies in PD [101]. Um et al. [102] showed that attachment of NEDD8 to Parkin increase the E3 ligase activity by increasing affinity towards E2 ubiquitin ligase UbcH8 and the putative substrate the p38 subunit of aminoacyl trasferase. Choo et al. [103] also reported increased Parkin E3 ligase activity upon neddylation. The also found that PINK1 undergoes neddylation, which results in increased stability of PINK1 55KDa proteolytic fragment. Interestingly, genetic enhancement of neddylation was shown to rescue the phenotypes associated with a *Drosophila* in vivo model of PINK1 deficiency. Moreover, PD neurotoxin MPP^+^ treatment showed decreased neddylation of both PINK1 and Parkin, clearly indicating a causal link between NEDD8 modification of PINK1/Parkin and PD pathogenesis.

## Nitrosylation, sulfhydration and sulfonation

Among many other contributing factors, nitric oxide (NO), hydrogen sulphide (H_2_S) and oxidative stress have been found to influence the progression of PD [104].

Increased attachment of NO to thiol groups (S-nitrosylation) of Parkin in PD was first reported by Ted Dawson’s group [105] where the authors showed increased nitosylation of Parkin in MPTP treated mice and human patient’s brains. They also demonstrated that nitrosylation decreased the protective effect of Parkin by inhibiting its E3 ligase activity. A concurrent study by Stuart Lipton’s group also reported the same while showing a steep increase of the Parkin E3 ubiquitin ligase activity, which autoubiquitinates the enzyme, followed by decreased activity [106].

In contrast to these works, one solitary study demonstrates that S-nitrosylation of Parkin, more specifically at Cys 323, increases the Parkin E3 ligase activity, and it is required for efficient removal of depolarised mitochondria [107]. The authors suggest that Cys 323 is not involved in zinc ion coordination and therefore its modification can regulate Parkin activity without disrupting the ability of the protein to coordinate ion zinc that is required for Parkin activity. Furthermore, the authors give evidences that PINK1 dependent phosphorylation and nitrosylation of Parkin act independently and that Parkin nitrosylation is mostly cytosolic.

These conflicting studies identified sites of potential S-nitrosylation within the RING1, RING2 and the IBR domain [106, 107]. Most of those that were identified as potential sites of nitrosylation are highly conserved among different species (except for Cys 323 that is only conserved in vertebrates), although further studies are required to understand whether these nitrosylated sites are responsible for altered Parkin E3 activity. Cysteine sites in these domains are important for zinc coordination and their nitrosylation is likely to distrupt the conformation of these domains that are both important for E2 coordination and Parkin catalytic activity. It is therefore not surprisingly that nitrosylation at those sites affect Parkin E3 ligase activity.

Interestingly, a recent work demonstrated that nitrosylation of Parkin increased p53 level [108]. Authors suggested that cell death due to nitrosative stress occurs via increase of pro apoptotic factor p53 and correlated the increased nitrosylation of Parkin to p53 levels in human post mortem PD brains. It was reported that nitrosylated Parkin preferentially accumulates in the cytoplasm and does not translocate to the nucleus, where Parkin operates as repressor of p53 promoter. This finding might explain the correlation between increased p53 levels and nitrosylated Parkin in PD brains, and potentially p53 dependent-cell death due to nitrosative stress, the later being the causative factor for the increase of p53 levels.

Modification of Parkin by H_2_S, termed sulfhydration, was found to be protective in nature. Three independent studies documented the protective effect of systemic administration of NaHS as H_2_S donor in preventing the progression of Parkinsonism in toxin induced animal models of PD [109–111]. In a subsequent study, Vaniver et al. [112], specifically discovered the sulfhydrated cysteine residues which enhanced Parkin protective activity. The authors systematically mutated the various Parkin cysteines and measured Parkin activity. Mutation C95S completely abolished Parkin enhanced ubiquitination activity upon administration of GYY4137, a H_2_S donor. Mutants C59S and C182S also result in substantial diminution of the enhancement of ubiquitination upon sulfhydration. Though the protective effect of sulfhydration against neurotoxin-induced Parkinsonism has been widely documented, the molecular mechanism is largely known.

Interestingly, the level of Parkin sulfhydration was found to be reduced in PD patient’s brain, while nitrosylation showed a steep increase. These findings suggest that nitosylation and sulfhydration are two reciprocal, opposing events that both impinge on cysteine residues and provide them with chemical groups that opposingly impact Parkin E3 ligase activity [112].

In a slightly different context, it was shown that heat shock, oxidative stress induced by H_2_O_2_ or deletion of 13 amino acids from the C terminal end of Parkin can lead to its aggregation, which is inhibited by the overexpression of heat shock protein chaperones [113]. More recently, a study by Meng et al. [114] showed that oxidative stress induced by either MPP+ or H_2_O_2_ treatment result in oxidation of a specific subset of cysteine residues of Parkin. The process of cysteine oxidation, also known as sulfonation, alters Parkin solubility and leads to Parkin inactivation. Upon mass spectrometry analysis of Parkin oxidation, authors also showed that several of the Parkin cysteine that are sulfonated upon oxidative stress (Cys 212, Cys 253, Cys 268, Cys 289, Cys 431 and Cys 441) were previously reported to be mutated in familial PD cases, supporting the hypothesis that rare hereditary mutations and environmentally linked PD cases might share a common mechanism of inactivation of Parkin.

## Post translational modifications of Parkin: a protein analysis between orthologs

In order to keep their function, proteins need to preserve their three-dimensional structure, meaning that they have to keep the same or similar amino acid sequence. If there are conserved amino acids in some regions of orthologous proteins, it can be concluded that these amino acids are important for the function of the protein. This is especially relevant when the conservation occurs at the protein rather than at the DNA level. Interestingly, amino acids sequence alignment between Parkin orthologs revealed that the Parkin residues that are post translationally modified are highly conserved from mammals to insects (Fig. 1a). These include sites of phosphorylation (Ubl and RING0 resident residues Ser 65, Tyr 175 and Tyr 217), sulfhydration (Ubl and RING0 resident residues Cys 59 and Cys 182) and sulfonation (RING1 and RING2 resident residues Cys 212, Cys 253, Cys 268, Cys 289, Cys 431 and Cys 441) (Fig. 1a).

It is interesting to note that phosphorylation sites leading to Parkin activation are highly conserved, whereas those which impairs Parkin activity are only conserved in mammals. It is intriguing to hypothesis that evolution might have been more stringent when it comes to mechanisms of Parkin activation.

This analysis also led us to the interesting observation that the residues that are post translationally modified to activate Parkin are either in the Ubl or the RING0 domain, whereas the inactivating modifications mainly affect residues of the RING1 or RING2 domain, that contain the E2 binding site and the catalytic site, respectively (Fig. 1c).

## Conclusions

Parkin is an E3 ubiquitin ligase with various pleiotropic functions. Elucidating the molecular mechanisms that control its function can have important implications not only in the regulation of mitochondria quality control and proteosomal dependent degradation of abnormal proteins, but also in the context of various Parkin cytoprotective functions, that include inhibition of the activity of pro apoptotic proteins p53 and Bax, and enhancement of expression of pro survival protein OPA1 via NF-kB signalling [29, 30].

Under basal conditions, Parkin adopts a coiled inhibited conformation and its activity is repressed [62–65, 115, 116]. Post translational modifications can control Parkin activity, subcellular localization, conformation, solubility, E2 choice and interaction with cofactors that are required for Parkin activation, substrate affinity as well as specificity. Post translational modifications can occur rapidly to respond to changes in metabolism or when cell experience environmental stress. More importantly, Post translational modifications are reversible and are controlled by the counteracting activities of opposing enzymes, which can be timely and rapidly regulated. Protein phosphatases oppose protein kinases, de-ubiquitinating enzymes oppose ubiquitin ligases, protein deacetylases counteract acetyltransferases, denitrosylation opposes S-nitrosylation and so on. Balanced regulation of opposing events can result in complex biological outcomes, particularly when the targets of this regulation are proteins with pleiotropic functions, like Parkin.

Potentially each PTM can be targeted for therapeutic intervention as long as its physiological outcomes is known and specific synthetic molecules are available to either inhibit or enhance such modification depending on its outcome. In this context, much effort has been recently put towards the identification of specific deubiquitinating enzymes that directly or indirectly oppose Parkin activity. Along the same line, it will be important to evaluate whether specific protein phosphatases are in place to oppose PINK1 in the phosphorylation of Parkin.

Future works will also clarify whether other modifications, such as acetylation (second most common PTM after phosphorylation) or glycosylation (third most common PTM) might play a role in the control of Parkin activation.

The next challenge will be to identify appropriate in vitro system that allows rapid and specific read out of Parkin activation. In that respect, in a very recent report Pao et al. [117] developed a newly engineered probe to monitor the thansthiolation activity of E3 ligases to decipher mechanisms of Parkin activation. Interestingly, this report demonstrates that initial phosphorylation of ubiquitin is upstream Parkin phosphorylation and subsequent activation, allowing the precise dissection of a rather complex multi step process.

The compatibility of newly generated probes to study Parkin activity from cell extracts and the potential reproducibility of the assay in samples extracted from human patients, will pave the way for the development of rapid methods to address how different post-translational modifications affect Parkin activity in vitro as well as in vivo.

## Reviewers’ comments

This article was reviewed by Prof. Dr. Konstanze F. Winklhofer, Faculty of Medicine, Biochemistry and Pathobiochemistry department, University of Ruhr-Bochumand (reviewer 1) and by Prof. Thomas Simmen, Department of Cell Biology, University of Alberta, Canada (reviewer 2).

**Reviewer 1 summary**: In their manuscript, the authors provide a comprehensive and timely review on the regulation of the E3 ubiquitin ligase Parkin by posttranslational modifications. This overview is well-balanced and includes a wide spectrum of Parkin functions. Therefore it will be interesting and helpful for a broad readership.

**Reviewer 2 summary**: The review article by Chakraborty et al. provides a useful, up-to-date resource for Parkin researchers by describing the complete set of known Parkin post-translational modifications. There is a three-part figure that accompanies the manuscript, which provides a helpful illustration of what is described within the manuscript. The sequence of chapters is logical, starting with phosphorylation. Ultimately, my suggestions are minor changes that would hopefully make the manuscript even more useful that it already is.

Response to reviewers: *First of all we would like to thank both reviewers for their valuable and relevant comments. As specified in the following chapters, reviewers concerns have been carefully addressed in the revised version of the manuscript*.

### Reviewer 1

Comment: Some suggestions to increase linguistic clarity: *Page 10, line 15 to 18: “Quite intriguingly, phosphorylation of Parkin by cyclin dependent kinase 5 (Cdk-5), casein kinase 1 (CK-1) and c-Abl leads to either increased Parkin aggregates properties or Parkin inhibition.” … modulates Parkin folding and/or activity*.

Response: *we have modified the sentence according to the suggestion*.

Comment: *Page 11, line 15: The statement that “mutation in the Ubl domain leads to multi ubiquitinated Parkin that is constitutively active” should be substantiated by a reference*.

Response: *We have modified the sentence and supported the statement by reference*.

Minor points: *Check correct spelling, for example:-Page 3, line 3: “familiar”-Page 4, line 15: “rational”-Page 8, line 15: “autoinhibitions”-Page 14, line 11: “S-nitrosylayion” - Fig.* *1* *a: “Sulfhydatin” Check wording, for example:-Page 3, line 4 : “brought to”-Page 5, line 21: “whether” - Page 14, line 10: “imposes” - Page 17, line 4: “whether”*

Response: *we have modified the specified sentences*.

### Reviewer 2

Comment: *what is the mechanism that causes proteasome degradation of mitochondrial proteins? Are these proteasomes mitochondria-localized? This is not well explained*.

Response: *we have explained this in the updated manuscript*.

Comment: *the description of highly conserved residues within Parkin on page 9 is confusing. Are all of these post-translationally modified? Are only these post-translationally modified?*

Response: *we have mentioned in the manuscript that most of these are post translationally modified. This list might not be exclusive and we have focused on the so far reported ones*.

Comment: *I would reconsider using the PTM abbreviation. I had some trouble getting used to it*.

Response: *to our knowledge, PTM is quite frequently used abbreviation for posttranslational modification. We however reconsidered using the abbreviation in the manuscript, as suggested by the reviewer*.

Comment: *on page 5, it is stated that PINK1 is a serine/threonine kinase, but on page 9, PINK1 is said to phosphorylate tyrosines on Parkin. It is later mentioned that Parkin serines are also phosphorylated. What is the relationship between tyrosine and serine phosphorylation? This needs to be better explained. What phosphorylation occurs in D. melanogaster? What is the relationship between phosphorylation by PINK1 and other kinases? What is known about Parkin serine phosphorylation in PD patient brains?*

Response: *We would like to thank the reviewer for actually spotting this. We realised that the abbreviation for Threonine was misspelled (Tyr instead of Thr): PINK1 indeed is a serine/threonine kinase that phosphorylates substrates on threonine (Thr) residues. We apologise for the confusion the misspelled abbreviation might have caused*.

*To our knowledge, nothing is known about Parkin serine phosphorylation in PD patients brains*.

Comment: *What is the functional difference between SUMO-1 and SUMO-2?*

Response: *The basic difference between SUMO-1 and 2 is their substrate preference. We have mentioned this in the manuscript*.

Comment: *Where could NO originate that modifies Parkin and why does it increase in PD brain? It is unclear whether there is a correlative or causative link between Parkin nitrosylation and p53*.

Response: *NO is synthesized by nitric oxide synthase (NOS), which comes in three forms: endothelial, neuronal and inducible NOS. So, in the brain the origin of NO could be due to the functionality of neuronal NOS. The increase in NO in PD brain and its relevance is quite controversial as it can be marked as both causative and/or after effect of neuro-inflammation. It is beyond the scope of this review to discuss that in details*.

*In the stated report the authors discovered that nitrosylation of Parkin decreases its activity as a repressor of P53. Later increase in both nitrosylated Parkin and P53 was found in human patients brain and the conclusion was drawn stating nitrosylated Parkin is the causal factor for P53 increase. We have mentioned this in the updated MS*.

Comment: *The authors may consider swapping panels a and b in Fig.* *1*.

Response: *We don’t understand the reason for this request. Panel a is described before panel b in the text*.

Comment: *English grammar and spelling needs to be improved in some spots. This is especially the case in the abstract, which I suggest to rewrite from scratch, but also in some other instances (e.g., lines 19–23 p. 5; line 18 p. 6; lines 11–13 p.9; lines 8–14 p. 11; lines 7, 13 p.12). Some issues with singular/plural confusion and wrong usage of articles*.

Response: *We have modified the manuscript according to the suggestions*.

## Abbreviations

Cdk-5: Cyclin dependent kinase 5
Ck-1: Casein kinase 1
DUBs: Deubiquitination enzymes
H_2_S: Hydrogen sulphide
IBR: In between RING finger domain
NO: Nitric oxide
OMM: Outer mitochondrial membrane
PD: Parkinson’s Disease
REP: Repressive element of Parkin
SUMO: Small ubiquitin like modifiers
UPS: Ubiquitin-proteasome system
